# Familial Longevity Is Marked by Lower Diurnal Salivary Cortisol Levels: The Leiden Longevity Study

**DOI:** 10.1371/journal.pone.0031166

**Published:** 2012-02-13

**Authors:** Raymond Noordam, Steffy W. M. Jansen, Abimbola A. Akintola, Nicole Y. L. Oei, Andrea B. Maier, Hanno Pijl, P. Eline Slagboom, Rudi G. J. Westendorp, Jeroen van der Grond, Anton J. M. de Craen, Diana van Heemst, On behalf of the Leiden Longevity Study group

**Affiliations:** 1 Department of Gerontology and Geriatrics, Leiden University Medical Center, Leiden, the Netherlands; 2 Department of Radiology, Leiden University Medical Center, Leiden, the Netherlands; 3 Department of Endocrinology, Leiden University Medical Center, Leiden, the Netherlands; 4 Section of Molecular Epidemiology, department of Medical Statistics and Bioinformatics, Leiden University Medical Center, Leiden, the Netherlands; 5 Leiden Institute for Brain and Cognition, Leiden, the Netherlands; 6 Netherlands Consortium of Healthy Ageing, the Netherlands; Universidad Europea de Madrid, Spain

## Abstract

**Background:**

Reported findings are inconsistent whether hypothalamic-pituitary-adrenal (HPA) signaling becomes hyperactive with increasing age, resulting in increasing levels of cortisol. Our previous research strongly suggests that offspring from long-lived families are biologically younger. In this study we assessed whether these offspring have a lower HPA axis activity, as measured by lower levels of cortisol and higher cortisol feedback sensitivity.

**Methods:**

Salivary cortisol levels were measured at four time points within the first hour upon awakening and at two time points in the evening in a cohort comprising 149 offspring and 154 partners from the Leiden Longevity Study. A dexamethasone suppression test was performed as a measure of cortisol feedback sensitivity. Age, gender and body mass index, smoking and disease history (type 2 diabetes and hypertension) were considered as possible confounding factors.

**Results:**

Salivary cortisol secretion was lower in offspring compared to partners in the morning (Area Under the Curve = 15.6 versus 17.1 nmol/L, respectively; p = 0.048) and in the evening (Area Under the Curve = 3.32 versus 3.82 nmol/L, respectively; p = 0.024). Salivary cortisol levels were not different after dexamethasone (0.5 mg) suppression between offspring and partners (4.82 versus 5.26 nmol/L, respectively; p = 0.28).

**Conclusion:**

Offspring of nonagenarian siblings are marked by a lower HPA axis activity (reflected by lower diurnal salivary cortisol levels), but not by a difference in cortisol feedback sensitivity. Further in-depth studies aimed at characterizing the HPA axis in offspring and partners are needed.

## Introduction

Cortisol secretion is tightly regulated by the hippocampus and the hypothalamic-pituitary-adrenal (HPA) axis through a negative feedback mechanism [1]. In the brain, binding of cortisol to high affinity mineralocorticoid receptors plays an important role in negative feedback control under basal conditions, while binding of cortisol to low affinity glucocorticoid receptors plays an important role in feedback control during stress. In healthy individuals, cortisol levels show a distinct rise directly after awakening, which reaches peak levels at 30 minutes and returns to baseline levels 60 minutes after awakening. Cortisol levels then gradually fall as the day progresses and reach a trough around midnight [2]. The distinct rise in cortisol levels upon awakening [3] is considered as a response to awakening (this distinct pattern is therefore also known as the cortisol awakening response or CAR), which is superimposed on the ultradian rhythm during the circadian cycle [4]. Because of its intra-individual stability, the cortisol awakening response is considered a trait measure for HPA axis activity [3].

Changes in HPA axis activity are associated with numerous pathophysiological conditions, for example persons under chronic stress or with depression have, on average, higher levels of cortisol [5], [6]. In addition, the cortisol awakening response is blunted or even absent in subjects having hippocampal damage, diabetes and hypertension [7], [8]. Higher evening cortisol levels (within normal physiological ranges) are associated with several clinical and physiological parameters, including a higher blood pressure and a more insulin resistant metabolic profile [9], [10], [11]. However, (cross-sectional) studies yielded inconsistent results regarding the changes that occur in HPA axis activity with increasing age. In some studies an increase in the cortisol awakening response was observed with increasing age [12], while others showed an opposite [13] or unaffected association [14]. Moreover, some studies showed an increase in cortisol levels in the evening with increasing age, while others showed no effect [12], [15]. Additionally, research showed that the HPA axis becomes less resilient in response to stress and becomes less sensitive to the negative feedback signals of glucocorticoids with increasing age [16]. In dogs, it was shown that hippocampal volume as well as the number of hippocampal mineralocorticoid receptors decrease with age [17]. These anatomical and functional changes are indicative of a reduced inhibition of the HPA axis, resulting in an increase in cortisol secretion.

Since the aforementioned studies compare young and old subjects, results from these studies might be confounded by a difference in prevalence of age-related diseases and depression. In the Leiden Longevity Study we have previously shown that middle aged offspring from long lived nonagenarian siblings seem biologically younger than their age and environmentally matched partners as reflected in a lower prevalence of age-related diseases [18], lower mortality [19], lower glucose levels [20], and higher insulin sensitivity [21], [22]. If diurnal cortisol levels increase with age, we would expect lower cortisol levels in these subjects compared to controls. To test this hypothesis, three research aims were addressed. First, saliva cortisol levels within the first hour upon awakening were measured as an assessment of the cortisol awakening response. Second, evening cortisol levels were assessed as an estimation of the lowest cortisol levels during the day. And third, a dexamethasone suppression test was performed to assess the cortisol feedback sensitivity. Measurements were performed in a random subpopulation from the Leiden Longevity Study comprising of 149 offspring and 154 partners.

## Materials and Methods

### Study design

The Leiden Longevity Study was designed to identify genetic and phenotypic markers related to longevity. A more detailed description of the recruitment strategy of the Leiden Longevity Study can be found elsewhere [23]. In short, a total of 421 families were recruited consisting of long-lived Caucasian siblings together with their offspring and partners thereof. The selection was based on the presence of at least two long-lived siblings that were still alive and fulfilled the age criteria of 89 years in case of males and 91 years for females [19], irrespective of health conditions and demographics. Because proper controls at high age are lacking, the offspring from these nonagenarian siblings were asked to participate and serve as cases, as they have an increased propensity to reach an old age. The partners of the offspring were asked to participate in the study as environmental- and age-matched controls.

For the present study 388 subjects (194 offspring and 194 partners) were enrolled from the Leiden Longevity Study. Saliva cortisol data were incomplete (at least one missing cortisol measurement out of the seven measurements or at least one missing time point of saliva collection) or invalid in 84 subjects (45 offspring and 39 partners) and these subjects were therefore excluded from the analyses. One subject (partner) used oral corticosteroids at the time of the study and was thus excluded from the analyses as well. None of the participants used inhaled corticosteroids. In total, data from 149 offspring from 93 families and 154 controls (their partners) were used for analyses. This study was approved by the Medical Ethical Committee of the Leiden University Medical Center and written informed consent was obtained from all participants.

### Salivary cortisol samples

Subjects were asked to collect saliva samples at home on an average weekday. Instructions for saliva sampling were given both orally (by a research nurse) and written. In total, six saliva samples for cortisol determination were obtained at one day. Four samples were taken at different time points in the first hour after awakening (at awakening and at 30 min, 45 min and one hour after awakening). These four time points were used to assess the CAR. The other two samples were taken in the evening, namely at 10 pm and 11 pm, as an estimation of the lowest cortisol levels during the day. For analyses, we refer to these samples to be taken at day “0”. After the last saliva sample (at 11 pm), subjects were asked to ingest one dexamethasone tablet (0.5 mg). At awakening the next day (day “1”), one additional saliva sample was taken for an estimation of the cortisol level after dexamethasone treatment.

### Salivary cortisol measurements

Measurements were performed using fully automatic equipment. Cortisol was measured using an Electrochemoluminescence Immunoassay (ECLIA) on the Modular E-170 Immunoanalyser (Roche Diagnostics, Mannheim, Germany). Coefficients of variance were below 5.7% in the morning and below 9.7% in the evening samples.

### Other variables

Additionally, weight and height were measured by research nurses at the study center. Information about current smoking habits was obtained using a questionnaire and information on disease history was obtained via the general practitioner and antidepressant drug use was obtained via the pharmacies.

### Statistical analyses

All data used for this study were normally distributed. The Area Under the Curve was calculated with respect to the ground (y = 0; cortisol levels were zero) as a measure for cortisol secretion in the first hour after awakening and for the evening time points (AUC_g_) [24]. To assess the increase in cortisol upon awakening during the CAR, we calculated the Area Under the Curve (AUC_i_) with respect to the level of cortisol at time point 0 [24]. To account for differences in time interval between individuals, both AUC's were divided by the time of saliva collection between the first and last time sample. As a measure of suppression by dexamethasone, the difference between the awakening salivary cortisol concentration on day 0 and the awakening salivary cortisol concentration after overnight dexamethasone treatment was calculated.

Analyses of the general characteristics were performed using student t test (continuous data) and chi square statistics (for categorical data). Analyses concerning the comparison between offspring and partners in salivary cortisol concentrations were performed using linear regression models. First, data are presented unadjusted for possible confounding factors. Second, data are presented after adjustment for the possible confounding factors: age, gender, body mass index and current smoking habits. Third, an additional analysis was performed in which adjustment was made for the history of type 2 diabetes and hypertension. Fourth, analyses were additionally adjusted for the use of antidepressant drugs, as these were previously described to associate with cortisol levels. Correlation between morning and evening cortisol levels was calculated using pearson chi square. The analysis concerning the dexamethasone suppression test was additionally adjusted for the cortisol salivary concentration at awakening at day 0 to account for possible differences observed at day 0 that might modify the results at day 1. In addition, an interaction term in the statistical model was used to assess whether the association between age and the AUC_g_ of the cortisol awakening response and cortisol levels in the evening, and the effectiveness of the dexamethasone tablet was similar in offspring and partners.

All statistical analyses were performed using SPSS for Windows (version 17.0, USA). In addition, analyses were adjusted for familial relationship using residual weight in SPSS. P-Values below 0.05 were considered statistically significant.

## Results

### Population characteristics

Characteristics of the study population are presented in table 1. The offspring and partner groups were similar with regard to the percentage of females, age, body mass index and smoking habits. Offspring had a lower prevalence of type- 2 diabetes compared to their partners, but were similar in the prevalence of the other diseases and the usage of antidepressant drugs.

**Table 1:** General characteristics of the study population.

	Offspring (n = 149)	Partners (n = 154)	P- Value
**Demographics**			
Females, no.(%)	72 (48.3)	84 (54.5)	0.28
Age (years)	66.0 (5.9)	65.5 (7.2)	0.57
Body Mass Index (kg/m2)	26.4 (3.9)	26.7 (4.2)	0.50
Current smokers, no.(%)	17 (11.5)	20 (13.2)	0.66
**Disease History, no.(%)**			
Type 2 Diabetes	4 (2.7)	15 (9.7)	0.010
Hypertension	32 (21.5)	41 (26.6)	0.27
Myocardial Infarction	1 (0.7)	3 (1.9)	0.32
Stroke	2 (1.3)	4 (2.6)	0.44
Rheumatoid arthritis	2 (1.3)	1 (0.6)	0.56
COPD	8 (5.4)	6 (3.9)	0.57
Antidepressant drugs, no.(%)	6 (4.0)	7 (4.5)	0.82

Age and body mass index are presented as means with the standard deviation. Abbreviation: COPD Chronic Obstructive Pulmonary Disease.

### Salivary cortisol levels

**Figure 1:**
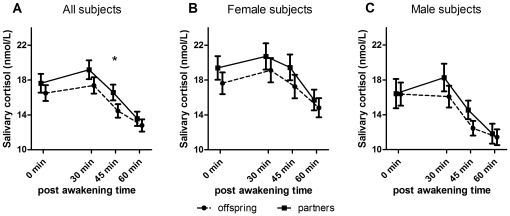
Awakening response in offspring and partners. Cortisol Awakening Response (CAR). All three graphs present the mean cortisol level measured at the four time points. A) CAR in all offspring and partners. Analysis adjusted for age, gender, body mass index and current smoking habits. B) CAR in female offspring and partners. C) CAR in male offspring and partners. B,C) analysis adjusted for age, body mass index and current smoking habits. Data presented as means with the standard error of the mean (SEM). Statistical significance (p<0.05) denoted as an asterisk.

A graphical presentation of the cortisol awakening response in offspring and partners is presented in figure 1A. The analyses were adjusted for age, gender, body mass index and current smoking habits. At all four time points mean salivary cortisol tended to be lower in offspring compared to their partners. A trend of lower salivary cortisol levels in offspring was present in both females and males (figure 1B en 1C).

To estimate the total cortisol secretion within the first hour on awakening, the AUC was calculated with respect to the ground as well as to the cortisol level on awakening. Results on total cortisol secretion on awakening are presented in table 2. After adjustment for the possible confounding factors, including gender, age, body mass index and current smoking, offspring had a nearly significant tendency toward a lower total cortisol secretion compared to their partners (AUCg = 15.9 vs 17.4 nmol/L, respectively; p = 0.051). After additional adjustment for disease history (type 2 diabetes and hypertension), the difference in AUC_g_ between offspring and partners persisted, and reached statistical significance (p = 0.048). Additionally, adjusting for antidepressant drug use did not materially change the difference in morning AUC_g_ between offspring and partners. Restriction to couples comprising an offspring and partner did not materially change the difference either. Furthermore, the AUC_i_ with saliva cortisol concentration at awakening as a reference was not different between offspring and partners (p = 0.66).

**Table 2:** Salivary cortisol in offspring and partners.

	Offspring (n = 149)	Partners (n = 154)	P - Value
**Morning (AUC_g_)**			
Model 1	15.4 (14.5–16.3)	16.7 (15.4–17.9)	0.12
Model 2	15.9 (14.5–17.2)	17.4 (15.9–18.9)	0.051
Model 3	15.6 (13.4–17.7)	17.1 (15.1–19.2)	0.048
**Evening (AUC_g_)**			
Model 1	3.21 (2.96–3.45)	3.68 (3.34–4.01)	0.027
Model 2	3.41 (3.05–3.76)	3.89 (3.47–4.30)	0.026
Model 3	3.32 (2.75–3.90)	3.82 (3.26–4.38)	0.024

Means presented as mean salivary cortisol in nmol/L with 95% confidence internal.

Model 1: Crude; Model 2: Adjusted for age, gender, body mass index and current smoking; Model 3: Adjusted for age, gender, body mass index, current smoking and disease history (Type 2 Diabetes and Hypertension). All analyses were adjusted for familial relationship. Data presented as means with 95% confidence interval. Abbreviation; AUC_g_ Area Under the Curve with reference to the ground.

**Figure 2:**
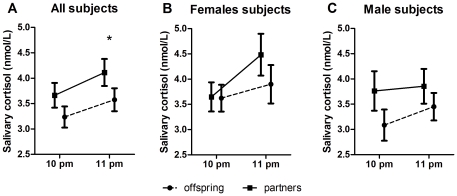
Evening cortisol in offspring and partners. Evening cortisol. All three graphs present the mean cortisol level measured at the two time points. A) Evening cortisol in all offspring and partners. Analysis adjusted for age, gender, body mass index and current smoking habits. B) Evening cortisol in female offspring and partners. C) Evening cortisol in male offspring and partners. B,C) analysis adjusted for age, body mass index and current smoking habits. Data presented as means with the standard error of the mean (SEM). Statistical significance (p<0.05) denoted as an asterisk.

Evening salivary cortisol was lower in offspring compared to their partners (figure 2A), which was significant at 11 pm (p = 0.045) and nearly significant at 10 pm (p = 0.078). On calculating the Area Under the Curve on the two time points (as a measure of the average cortisol levels per hour in the evening; lower half of table 2), offspring had lower salivary cortisol levels compared to their partners (3.41 vs 3.89 nmol/L; p = 0.026). The differences in evening AUC_g_ between offspring and partners were similar when the analysis was additionally adjusted for disease history as well as when antidepressant drug use was included as a potential confounding factor. The trend towards lower evening cortisol levels in offspring was similar in females and males (figure 2B and C).

The difference between offspring and partners persisted when the analysis was restricted to a subsample comprising only couples of offspring and partners.

The AUC of the cortisol awakening response and the AUC of the evening cortisol were positively correlated to each other (Pearson Correlation, r = 0.25, p = <0.001). The association between the AUC of the cortisol awakening response and the AUC of the evening cortisol was similar for offspring and partners (p for interaction = 0.51, when adjusted for age, gender, body mass index, current smoking and disease history).

### Dexamethasone suppression test

Results of the overnight dexamethasone test are presented in table 3. After adjusting for possible confounding factors, awakening salivary cortisol levels after dexamethasone treatment were not different between offspring and partners (4.82 vs 5.26 nmol/L, respectively; p = 0.26). Moreover, adjustment for the salivary cortisol concentration at awakening on day 0 did not materially change this analysis. Furthermore, the difference between salivary cortisol levels at awakening with and without dexamethasone, as a measure of effectiveness, was similar for offspring and partners (10.0 vs 11.0 nmol/L; p = 0.37). The association between age and the effectiveness of the dexamethasone tablet was similar for offspring and partners (p for interaction = 0.63 when adjusted for age, gender, body mass index, current smoking and disease history).

**Table 3:** Salivary cortisol levels after overnight dexamethasone.

	Offspring (n = 149)	Partners (n = 154)	P - Value
**Salivary cortisol level^1^**			
Model 1:	4.34 (3.89–4.80)	4.79 (4.16–5.42)	0.26
Model 2:	4.75 (4.09–5.42)	5.21 (4.43–6.00)	0.25
Model 3:	4.82 (3.76–5.88)	5.26 (4.21–6.31)	0.28
Model 4:	4.94 (3.91–5.97)	5.26 (4.24–6.28)	0.43
**Difference levels^2^**			
Model 1:	11.8 (10.6–13.1)	11.9 (10.2–13.6)	0.94
Model 2:	11.6 (9.8–13.4)	12.3 (10.2–14.4)	0.50
Model 3:	10.0 (7.2–12.9)	11.0 (8.2–13.8)	0.37

Means presented as mean salivary cortisol in nmol/L with 95% confidence internal. Model 1: Crude; Model 2: Adjusted for age, gender, body mass index and current smoking; Model 3: Adjusted for age, gender, body mass index, current smoking and disease history (Type 2 Diabetes and Hypertension); Model 4: Adjusted for age, gender, body mass index, current smoking and disease history (Type 2 Diabetes and Hypertension) and salivary cortisol concentration at awakening on the first day. All analyses were adjusted for familial relationships. 1) Awakening salivary cortisol levels after overnight dexamethasone. 2) Difference between salivary cortisol level on awakening at day 0 and overnight dexamethasone.

## Discussion

In this study we aimed to investigate cortisol levels and cortisol feedback sensitivity in relation to familial longevity. We first showed that salivary cortisol levels were lower in the offspring compared to their partners both in the morning and in the evening. Second, we showed that cortisol feedback sensitivity, as estimated by a dexamethasone suppression test, was similar between offspring and partners, suggesting that the difference between offspring and partners in salivary cortisol concentration is most likely not caused by a difference in cortisol feedback sensitivity.

### Cortisol levels and familial longevity

Previous research showed that, when comparing young and old subjects, cortisol levels were higher during the night in older subjects compared to younger subjects [12], [15]. One of the possible reasons for the increase in cortisol is the neuronal loss in the hippocampus resulting in an impaired negative feedback mechanism [14]. Research on twins suggests a substantial heritability of 62% on cortisol levels, suggesting a genetic contribution [25]. Compared to their partners, the offspring have a lower incidence of age-related diseases [18], rate of mortality [19] and serum glucose [20] and higher insulin sensitivity [21], [22], suggesting that they are biologically younger. Therefore, the results of the present study might suggest that in middle aged individuals low HPA activity marks better metabolic/cardiovascular health and a lower biological age. In addition, as cortisol was shown to be associated with insulin resistance [9], this study might suggest that the enhanced insulin sensitivity in the offspring might be facilitated by the lower cortisol levels. After adjustment for the two most prevalent age-related diseases (hypertension and type- 2 diabetes), the difference in cortisol levels between offspring and partners persisted, suggesting that is not caused by a difference in disease prevalence.

The cortisol awakening response is dependent on both the circadian rhythm as well as on awakening itself [4]. Interestingly, the increase upon awakening (estimated by the AUC_i_), was similar in both groups. Because the increase upon awakening was similar in both groups and because the offspring had lower levels of cortisol in both the morning and evening it might be suggested that offspring have lower total cortisol secretion compared to their partners.

Socio- economic status is described to influence the cortisol awakening response also [26]. Persons with a low socio-economic status have higher levels of cortisol in the morning compared to persons with a high status. In this study, we compared offspring from nonagenarian siblings with environmentally matched controls (their partners). It is therefore unlikely that the difference between the offspring and partners is confounded by social- economic status.

### Cortisol sensitivity and familial longevity

With increasing age, sensitivity to cortisol is decreasing. A recent study showed that increasing the dexamethasone dose has no additional effect on inhibiting cortisol secretion in elderly, whereas it does in younger subjects [16]. These results indicate that the negative feedback on the HPA axis is becoming less effective. One of the mechanisms described is the decreased expression of mineralocorticoid receptors in the hippocampus with increasing age [17]. In the present study, we showed that cortisol levels after overnight dexamethasone and the effectiveness of dexamethasone were similar in offspring and partners. These results indicate that cortisol sensitivity is similar in both study groups. The lower salivary cortisol levels in the offspring therefore seem not to be due to a difference in cortisol sensitivity. The dose used in this study (0.5 mg) is used for clinical purposes only. As the present study was performed in healthy individuals, the difference in responses may be clearer when a lower dose of dexamethasone was used. Repetition of this experiment with a lower dexamethasone dose might further elucidate whether cortisol feedback sensitivity is associated with familial longevity.

### Study limitations

This study has a few limitations to address. As this study was performed home-based, strict standardization and timing by which the saliva samples were taken cannot be ascertained. Additionally, we observed an increase in cortisol levels at 11 pm compared to 10 pm, in which a decrease was expected. A possible explanation could be that subjects who normally slept earlier were subjected to stress when asked to take saliva samples at 11 p.m. (thereby increasing their cortisol level). A third limitation of this study was that we did not have information on depression at the time of the study. Instead, we used information from the pharmacies on antidepressant drug use. The limitation of this strategy is that also participants with, for example, neuropathic pain and anxiety disorders might take antidepressant medication. However, as the number of participants taking antidepressant medication is small, the effect will likely be negligible. Despite these shortcomings, which probably resulted in an increased variation in the dataset, we were still able to demonstrate lower salivary cortisol levels in offspring from long-lived families both in the morning and evening.

### Conclusion

In conclusion, the results of this study show that familial longevity might be marked by lower levels of morning and evening cortisol. Dexamethasone suppression tests revealed that it is unlikely that a major change in cortisol feedback sensitivity explains these effects, although a repetition with a lower dose is recommendable. This study describes an association between cortisol levels and familial longevity. More research should be performed to characterize what mechanism is responsible for the lower levels of cortisol in the offspring group. Moreover, more research and replication with increased precision and standardization should be performed to further characterize the differences in HPA axis activity between longevity families and controls.

## References

[1] Beyer HS, Matta SG, Sharp BM (1988). Regulation of the messenger ribonucleic acid for corticotropin-releasing factor in the paraventricular nucleus and other brain sites of the rat.. Endocrinology.

[2] Kirschbaum C, Hellhammer DH (1989). Salivary cortisol in psychobiological research: an overview.. Neuropsychobiology.

[3] Pruessner JC, Wolf OT, Hellhammer DH, Buske-Kirschbaum A, von Auer K (1997). Free cortisol levels after awakening: a reliable biological marker for the assessment of adrenocortical activity.. Life Sci.

[4] Wilhelm I, Born J, Kudielka BM, Schlotz W, Wust S (2007). Is the cortisol awakening rise a response to awakening?. Psychoneuroendocrinology.

[5] Pruessner M, Hellhammer DH, Pruessner JC, Lupien SJ (2003). Self-reported depressive symptoms and stress levels in healthy young men: associations with the cortisol response to awakening.. Psychosom Med.

[6] Tafet GE, Idoyaga-Vargas VP, Abulafia DP, Calandria JM, Roffman SS (2001). Correlation between cortisol level and serotonin uptake in patients with chronic stress and depression.. Cogn Affect Behav Neurosci.

[7] Bruehl H, Wolf OT, Convit A (2009). A blunted cortisol awakening response and hippocampal atrophy in type 2 diabetes mellitus.. Psychoneuroendocrinology.

[8] Wirtz PH, von Kanel R, Emini L, Ruedisueli K, Groessbauer S (2007). Evidence for altered hypothalamus-pituitary-adrenal axis functioning in systemic hypertension: blunted cortisol response to awakening and lower negative feedback sensitivity.. Psychoneuroendocrinology.

[9] Phillips DI, Barker DJ, Fall CH, Seckl JR, Whorwood CB (1998). Elevated plasma cortisol concentrations: a link between low birth weight and the insulin resistance syndrome?. J Clin Endocrinol Metab.

[10] Walker BR, Phillips DI, Noon JP, Panarelli M, Andrew R (1998). Increased glucocorticoid activity in men with cardiovascular risk factors.. Hypertension.

[11] Walker BR, Soderberg S, Lindahl B, Olsson T (2000). Independent effects of obesity and cortisol in predicting cardiovascular risk factors in men and women.. J Intern Med.

[12] Van Cauter E, Leproult R, Kupfer DJ (1996). Effects of gender and age on the levels and circadian rhythmicity of plasma cortisol.. J Clin Endocrinol Metab.

[13] Knoops AJ, van der Graaf Y, Mali WP, Geerlings MI (2010). Age-related changes in hypothalamic-pituitary-adrenal axis activity in patients with manifest arterial disease.. Endocrine.

[14] Heaney JL, Phillips AC, Carroll D (2011). Ageing, physical function, and the diurnal rhythms of cortisol and dehydroepiandrosterone.. Psychoneuroendocrinology.

[15] Dodt C, Theine KJ, Uthgenannt D, Born J, Fehm HL (1994). Basal secretory activity of the hypothalamo-pituitary-adrenocortical axis is enhanced in healthy elderly. An assessment during undisturbed night-time sleep.. Eur J Endocrinol.

[16] Hatzinger M, Brand S, Herzig N, Holsboer-Trachsler E (2011). In healthy young and elderly adults, hypothalamic-pituitary-adrenocortical axis reactivity (HPA AR) varies with increasing pharmacological challenge and with age, but not with gender.. J Psychiatr Res.

[17] Rothuizen J, Reul JM, van Sluijs FJ, Mol JA, Rijnberk A (1993). Increased neuroendocrine reactivity and decreased brain mineralocorticoid receptor-binding capacity in aged dogs.. Endocrinology.

[18] Westendorp RG, van Heemst D, Rozing MP, Frolich M, Mooijaart SP (2009). Nonagenarian siblings and their offspring display lower risk of mortality and morbidity than sporadic nonagenarians: The Leiden Longevity Study.. J Am Geriatr Soc.

[19] Schoenmaker M, de Craen AJ, de Meijer PH, Beekman M, Blauw GJ (2006). Evidence of genetic enrichment for exceptional survival using a family approach: the Leiden Longevity Study.. Eur J Hum Genet.

[20] Rozing MP, Westendorp RG, Frolich M, de Craen AJ, Beekman M (2009). Human insulin/IGF-1 and familial longevity at middle age.. Aging (AlbanyNY).

[21] Rozing MP, Westendorp RG, de Craen AJ, Frolich M, de Goeij MC (2010). Favorable glucose tolerance and lower prevalence of metabolic syndrome in offspring without diabetes mellitus of nonagenarian siblings: the Leiden longevity study.. J Am Geriatr Soc.

[22] Wijsman CA, Rozing MP, Streefland TC, le Cessie S, Mooijaart SP (2011). Familial longevity is marked by enhanced insulin sensitivity.. Aging Cell.

[23] Schoenmaker M, de Craen AJ, de Meijer PH, Beekman M, Blauw GJ (2006). Evidence of genetic enrichment for exceptional survival using a family approach: the Leiden Longevity Study.. Eur J Hum Genet.

[24] Pruessner JC, Kirschbaum C, Meinlschmid G, Hellhammer DH (2003). Two formulas for computation of the area under the curve represent measures of total hormone concentration versus time-dependent change.. Psychoneuroendocrinology.

[25] Bartels M, Van den Berg M, Sluyter F, Boomsma DI, de Geus EJ (2003). Heritability of cortisol levels: review and simultaneous analysis of twin studies.. Psychoneuroendocrinology.

[26] Miller GE, Chen E, Fok AK, Walker H, Lim A (2009). Low early-life social class leaves a biological residue manifested by decreased glucocorticoid and increased proinflammatory signaling.. Proc Natl Acad Sci U S A.

